# The invisible emergencies: metabolic and neuro-critical disorders often missed in the pediatric ED — a narrative review

**DOI:** 10.1186/s12245-026-01186-1

**Published:** 2026-03-31

**Authors:** Eslam Abady, Karrar Naeem Karam, Sayyid Qasim Khaleel, Kayleigh Kuhn, Panos Tamvakologos, Kevin Thomas Mathew

**Affiliations:** 1 Faculty of Medicine, Tanta University, Tanta, Egypt; 2 Faculty of Medicine, Mansoura University, Mansoura, Egypt; 3 Department of Pediatrics, The Brooklyn Hospital Center, Brooklyn, NY USA; 4 St. George’s University School of Medicine, West Indies, Grenada; 5 David Tvildiani Medical University, Tbilisi, Georgia

**Keywords:** Pediatric emergency, Metabolic disorders, Neuro-critical care, Inborn errors of metabolism, Encephalopathy, Seizures, Early diagnosis, Stabilization

## Abstract

**Background:**

Delayed recognition of metabolic and neuro-critical disorders in children presenting to emergency departments (EDs) contributes significantly to morbidity and poor outcomes. Early symptoms are often subtle or nonspecific, mimicking common illnesses and leading to missed or delayed diagnoses. This review synthesizes recent evidence to identify frequently overlooked high-risk conditions and evaluate strategies to improve early identification, stabilization, and outcomes.

**Main body:**

This review identifies several categories of under-recognized pediatric emergencies. Metabolic crises often result from inborn errors of metabolism (IEMs), including urea cycle defects, organic acidemias, mitochondrial disorders, and fatty acid oxidation disorders, which can present with nonspecific findings like vomiting and lethargy. Neuro-critical emergencies frequently missed include nonconvulsive status epilepticus (NCSE), autoimmune encephalitis, acute disseminated encephalomyelitis (ADEM), and early signs of elevated intracranial pressure (ICP) from infection, trauma, or hydrocephalus. Key contributors to delayed diagnosis are the absence of focal deficits, low clinical suspicion for rare disorders, and the underutilization of rapid diagnostic tools. Consequences of delay range from metabolic decompensation to irreversible neurologic injury. Evidence-based strategies to enhance care include the implementation of red-flag-triggered testing algorithms (e.g., point-of-care ammonia, glucose, lactate, and ketones), early initiation of empiric metabolic protocols, and the expanded use of bedside tools such as point-of-care ultrasound (optic nerve sheath diameter) and continuous EEG where available. Furthermore, integrating clinical decision support into electronic health records and providing focused ED training modules have proven effective in reducing time-to-diagnosis.

**Conclusion:**

Enhancing the early recognition of pediatric metabolic and neuro-critical emergencies in the ED requires a multifaceted approach. Systematic implementation of evidence-based protocols, greater availability and use of point-of-care diagnostics, and improved interdisciplinary collaboration are essential to reduce diagnostic delays, prevent morbidity, and improve patient outcomes.

## Introduction

Children presenting to the emergency department (ED) with altered mental status, seizures, or unexplained deterioration pose a diagnostic challenge. While infectious encephalitis and structural causes (trauma, intracranial hemorrhage, tumors, hydrocephalus) are often considered first, metabolic and neuro-critical disorders—including inborn errors of metabolism (IEMs), status epilepticus secondary to metabolic encephalopathy, and mitochondrial crises—are frequently under-recognized and may be “invisible” unless specifically sought. Key metabolic triggers for status epilepticus include hypoglycemia, hyperammonemia, electrolyte disturbances, and IEM decompensation. Early recognition is critical because rapid, targeted interventions can reverse metabolic derangement and prevent irreversible neurologic injury [1–3].

**Fig. 1:**
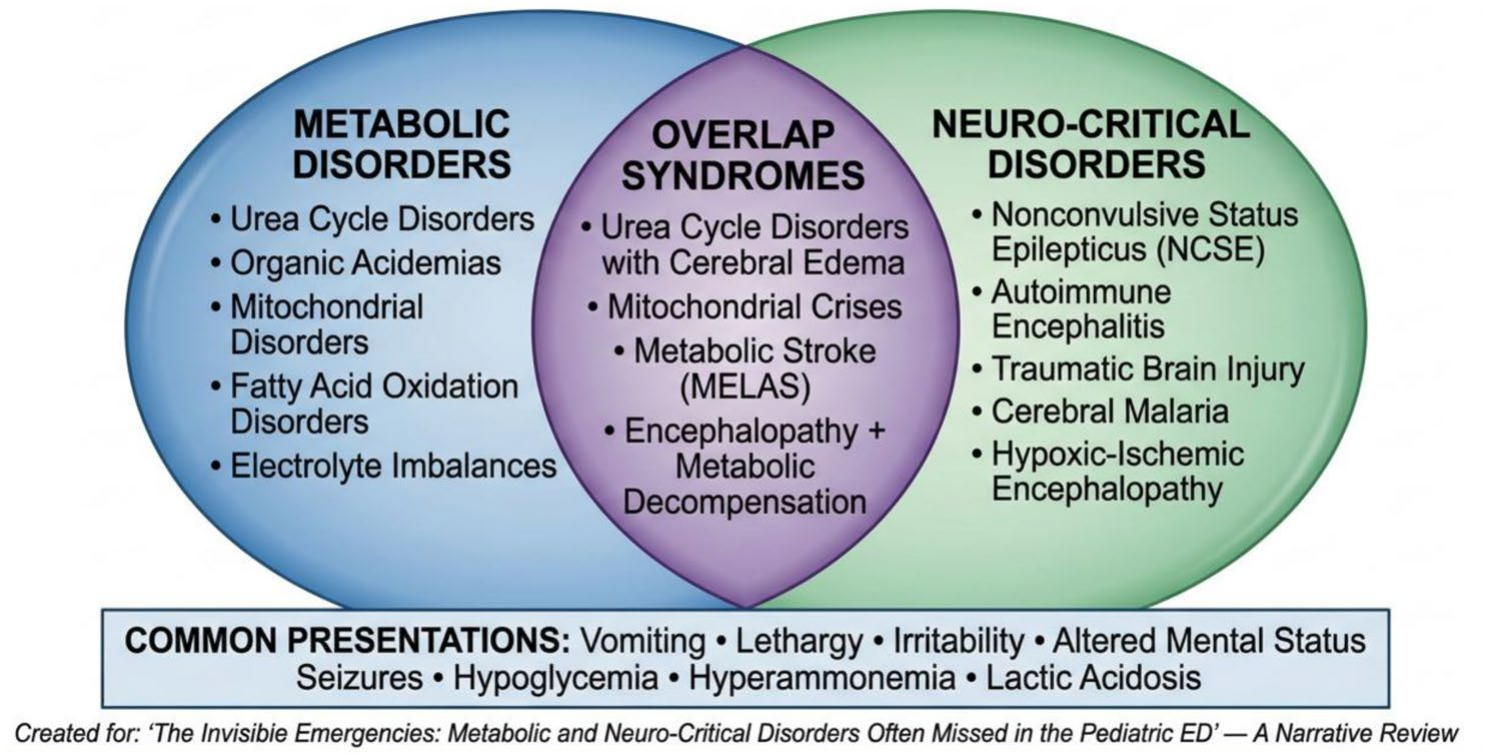
Conceptual diagram of “invisible emergencies” in the pediatric ED: overlap of metabolic and neuro-critical disorders, common presentations, and diagnostic challenges

The term **“invisible emergencies”** refers to life-threatening metabolic and neuro-critical conditions that mimic benign disorders due to nonspecific presentations such as lethargy, vomiting, irritability, or subtle seizures (e.g., eye deviation, nystagmus, autonomic changes without overt convulsions). In pediatric EDs, these disorders are often missed, leading to diagnostic delays, increased morbidity, and preventable mortality. Studies report that a substantial proportion—ranging from 30 to 50% depending on the condition and population studied—of pediatric metabolic crises present with nonspecific symptoms, and ED recognition time strongly predicts neurologic outcome [4, 5]. Global data highlight significant disparities in diagnostic capabilities and outcomes between low- and middle-income countries (LMICs) and high-income countries (HICs), underscoring the need for multidisciplinary awareness and structured approaches [6]. See Fig. 1.

As a narrative review, this manuscript represents a selective synthesis of relevant literature and clinical guidance rather than a systematic review, aiming to provide an educational overview and practical framework for ED clinicians. This review is structured as follows: classification of invisible emergencies (section “Classification of invisible emergencies”), detailed discussion of metabolic (section “Metabolic emergencies in the ED”) and neuro-critical (section “Neuro-critical emergencies in the ED”) emergencies, overlap syndromes (section “Overlapping syndromes”), diagnostic algorithms (section “Diagnostic algorithms”), management and stabilization (section “Management and stabilization”), LMIC considerations (section “Challenges in low- and middle-income countries (LMICs)”), a proposed clinical framework (section “Proposed clinical framework”), and future directions (section “Future directions and research gaps”).

### Search strategy and methodology

For this narrative review, we conducted a literature search of PubMed/MEDLINE, Scopus, and Web of Science databases from January 2000 to December 2024 using combinations of keywords including: “pediatric emergency,” “metabolic disorders,” “inborn errors of metabolism,” “neuro-critical care,” “encephalopathy,” “seizures,” “status epilepticus,” “hyperammonemia,” and “lactic acidosis.” We included English-language articles, guidelines, systematic reviews, and original research relevant to emergency department recognition and management. Reference lists of identified articles were manually screened for additional sources. Given the narrative format, no formal quality assessment or meta-analysis was performed; however, we prioritized evidence from high-impact journals, recent systematic reviews, and consensus guidelines where available. We have endeavored to follow SANRA (Scale for the Assessment of Narrative Review Articles) guidelines in structuring this review.

## Classification of invisible emergencies

Disorders encompassed within the concept of “invisible emergencies”—those metabolic and neuro-critical conditions frequently missed in pediatric EDs—can be classified based on their underlying pathogenesis into those driven by inborn errors of metabolism and those resulting from acute neuro-critical insults, though significant overlap exists in their clinical presentation and management [7].

### Metabolic pathogenesis

Disorders with a metabolic pathogenesis, collectively known as inborn errors of metabolism (IEMs), arise from genetic defects in enzymes, cofactors, or transporters that disrupt specific biochemical pathways [8, 9]. These conditions are generally classified into small-molecule and complex-molecule disorders [10]. Small-molecule defects often manifest as intoxication syndromes such as urea cycle disorders, maple syrup urine disease (MSUD), and organic acidemias, where toxic compounds accumulate proximal to a metabolic block [8–10]. These disorders typically present with acute encephalopathy, vomiting, and lethargy following a symptom-free interval, often precipitated by catabolic stressors like fever, infection, or fasting [8]. Alternatively, small-molecule defects may present as energy deficiency disorders such as mitochondrial diseases and fatty acid oxidation disorders (FAODs), characterized by a failure in production or utilization of energy substrates; these often present without a symptom-free interval and involve high-energy organs leading to hypoglycemia, cardiomyopathy, or myopathy [8]. Complex-molecule disorders, including lysosomal diseases and peroxisomal disorders, involve defects in the synthesis or catabolism of large molecules, leading to progressive neurodegeneration, structural brain malformations, and systemic signs like hepatosplenomegaly or dysmorphology [8]. The underlying mechanisms of brain injury in IEMs include cellular energy deprivation, direct neurotoxicity from accumulated metabolites (e.g., ammonia, leucine), and disruption of cell signaling or membrane integrity [11, 12].

### Neuro-critical pathogenesis

Disorders with a neuro-critical pathogenesis result from acute acquired insults such as traumatic brain injury (TBI), stroke, hypoxic-ischemic encephalopathy (HIE), or severe infections like cerebral malaria [13]. The pathophysiology is defined by a primary insult that triggers secondary injury mechanisms, including excitotoxicity, inflammation, hypoxia, and intracranial hypertension [14]. A central phenomenon in some neuro-critical conditions is the cerebral metabolic energy crisis (CMEC), identified by an elevated cerebrospinal fluid lactate-to-pyruvate ratio (LPR) [13]. The concept of CMEC, while extensively studied in pediatric cerebral malaria, may represent a common final pathway in multiple neuro-critical conditions including TBI, HIE, and post-cardiac arrest syndrome, though the evidence base varies across conditions [13, 14]. Management in the neuro-critical setting focuses on mitigating these secondary injuries by optimizing cerebral perfusion pressure, monitoring brain tissue oxygenation, and controlling intracranial pressure rather than specific metabolite dietary management used in IEMs [14].

#### Overlap syndromes

Certain IEMs, particularly “intoxication” type disorders such as urea cycle disorders (UCDs) and MSUD, represent a critical overlap between metabolic and neuro-critical areas [15, 16]. These disorders can lead to acute, life-threatening encephalopathies caused by specific biochemical accumulations. In UCDs, like ornithine transcarbamylase (OTC) deficiency, the inability to detoxify nitrogen results in rapid hyperammonemia [15, 16]. Excess ammonia is converted into glutamine, an osmotically active substance that accumulates in astrocytes, causing cytotoxic cerebral edema [15, 16]. Neuroimaging often shows a central pattern of edema affecting the peri-rolandic and basal ganglia regions while sparing the thalami, useful in differentiating UCDs from hypoxic-ischemic encephalopathy [16]. Similarly, in MSUD, the buildup of leucine and alpha-ketoacids results in diffuse intramyelinic edema and brain swelling [16]. These metabolic crises usually present in neonates or infants with sudden onset symptoms such as lethargy, vomiting, seizures, and coma, often after a symptom-free period [12, 17], mimicking conditions like sepsis or primary structural brain injury, underscoring the need for immediate neuro-critical identification to prevent cerebral herniation and irreversible neuronal damage [12, 17].

### Age of onset and differential diagnosis

In the ED, the age of symptom onset is crucial for narrowing the differential diagnosis between IEMs and other neurological conditions [3, 8]. For neonates, a key differentiating factor is the presence of a symptom-free interval [3, 8]. Intoxication disorders, such as UCDs, MSUD, and organic acidemias, typically present with acute encephalopathy after a brief period of normalcy, during which toxic metabolites accumulate [3, 8]. In contrast, energy deficiency disorders, like mitochondrial diseases and FAODs, often appear without a symptom-free interval and may involve multi-organ dysfunction, such as cardiomyopathy or hepatomegaly [3, 17]. Specific etiologies of neonatal seizures tend to present at characteristic ages, and the timing of onset therefore helps narrow the differential diagnosis [12, 18]. For instance, pyridoxine-dependent epilepsy and non-ketotic hyperglycinemia (NKH) typically present with intractable seizures within the first few days to weeks of life [12, 18].

As children grow from early childhood into adolescence, the differential diagnosis shifts towards intermittent metabolic presentations and acquired conditions [3]. While UCDs and organic acidemias are most common in newborns, they can also present in later childhood with symptoms like intermittent vomiting, ataxia, or psychiatric issues, often triggered by catabolic stress or high protein intake [3]. Conversely, disorders of metal metabolism, such as Wilson disease, typically manifest between ages 3 and 50, with symptoms including movement disorders or psychiatric decline, rather than acute neonatal encephalopathy [16, 19]. The presentation of acute acquired injuries varies by age as well. For example, traumatic brain injuries in toddlers are often caused by falls or non-accidental trauma (NAT), while motor vehicle accidents are the predominant cause in adolescents [14]. In toddlers presenting with TBI, NAT must be considered. Conversely, certain metabolic and genetic conditions (e.g., glutaric aciduria type I, Menkes disease) can predispose to subdural hemorrhages and mimic NAT, underscoring the need for metabolic evaluation in unexplained cases [3, 16]. Additionally, seizures are a common symptom of stroke in infants and children under 4 years of age, whereas older children are more likely to present with focal neurological deficits [14]. HIE can occur at any age following cardiac arrest, asphyxia, or severe hypotension, though it is most commonly discussed in the neonatal context. See Table 1.

**Table 1:** Classification and key features of invisible emergencies [14–19]

Disorder / Condition	Primary Category	Key Clinical Features	Typical Age at Presentation
Urea Cycle Disorders	Metabolic / Overlap	Acute encephalopathy, hyperammonemia, respiratory alkalosis, cerebral edema	Newborns (severe), any age (partial defects)
Maple Syrup Urine Disease	Metabolic	Encephalopathy, maple syrup odor, elevated BCAAs, intramyelinic edema	Newborns (classic), infancy (intermittent)
Organic Acidemias	Metabolic	Metabolic acidosis, ketosis, hyperammonemia, vomiting, lethargy	Newborns to childhood
Fatty Acid Oxidation Disorders	Metabolic	Hypoketotic hypoglycemia, hepatomegaly, cardiomyopathy, triggered by fasting	Infancy to adulthood
Mitochondrial Diseases	Metabolic / Overlap	Lactic acidosis, stroke-like episodes, encephalopathy, multiorgan involvement	Infancy to adulthood
Nonconvulsive Status Epilepticus	Neuro-Critical	Altered consciousness, subtle motor signs, diagnosed only by EEG	Any pediatric age
Autoimmune Encephalitis	Neuro-Critical	Psychiatric symptoms, seizures, movement disorders, often with autoantibodies	School-age to adolescents
Traumatic Brain Injury	Neuro-Critical	History of trauma, altered mental status, signs of ↑ICP, focal deficits	Toddlers (falls, NAT), adolescents (MVA)
Cerebral Malaria	Neuro-Critical	Coma, seizures, cerebral edema, microvascular obstruction, CMEC	6 months – 12 years
Hypoxic-Ischemic Encephalopathy	Neuro-Critical / Overlap	Perinatal or post-arrest insult, seizures, hypotonia, multi-organ dysfunction	Any pediatric age (neonates most common)

**Abbreviations**: BCAAs = branched-chain amino acids; EEG = electroencephalogram; ↑ICP = increased intracranial pressure; MVA = motor vehicle accident; CMEC = cerebral metabolic energy crisis; NAT = non-accidental trauma

## Metabolic emergencies in the ED

Metabolic emergencies in children often present with subtle, nonspecific symptoms that can rapidly escalate to life-threatening conditions if not recognized and treated promptly. Key metabolic crises include hypoglycemia, hyperammonemia, lactic acidosis, inborn errors of metabolism (IEMs), and electrolyte imbalances. Early point-of-care recognition and intervention within the first 60 min are critical to preventing irreversible neurologic injury [20, 21].

### Hypoglycemia

Hypoglycemia is a common and dangerous metabolic disturbance in pediatric patients, defined as blood glucose < 50–60 mg/dL (< 2.6–3.3 mmol/L). It can result from diverse etiologies including sepsis, hyperinsulinism, glycogen storage disorders, fatty acid oxidation defects, and adrenal insufficiency. Adrenal insufficiency should be considered in children with refractory hypoglycemia, hyponatremia, or shock, particularly those with a history of steroid use or suggestive skin findings (hyperpigmentation) [22]. In the ED, point-of-care glucose testing should be performed immediately in any child with altered mental status, seizures, or lethargy. Empiric treatment with intravenous dextrose (2–5 mL/kg of 10% dextrose) is lifesaving while awaiting confirmatory tests. Delayed correction can lead to hippocampal injury, long-term cognitive deficits, and death [23, 24].

### Hyperammonemia

Hyperammonemia, often defined as ammonia > 150–200 µg/dL in older children and > 200–400 µg/dL in neonates, is a medical emergency commonly caused by urea cycle disorders (UCDs), organic acidemias, or liver failure. Toxic ingestions (e.g., valproate, salicylates) should also be considered in the differential diagnosis, particularly in older children with acute onset and no prior metabolic history [25]. Hyperammonemia presents with progressive encephalopathy, vomiting, and respiratory alkalosis. Point-of-care ammonia testing should be obtained in any child with unexplained altered consciousness. Ammonia levels are highly labile; specimens must be collected without tourniquet stasis, placed immediately on ice, and transported rapidly to the laboratory for processing. Delayed processing or improper handling can result in falsely elevated levels, leading to unnecessary interventions [25, 26]. Immediate management includes protein restriction, intravenous dextrose to reduce catabolism, nitrogen scavengers (sodium benzoate, sodium phenylacetate), and prompt referral for dialysis (e.g., continuous veno-venous hemodiafiltration) if levels exceed 500 µg/dL or if clinical deterioration occurs [25, 26].

### Lactic acidosis

Lactic acidosis (blood lactate > 2–4 mmol/L) may indicate mitochondrial disorders, sepsis, hypoperfusion, or intoxication-type IEMs. Elevated lactate with a high lactate-to-pyruvate ratio (> 20–25) suggests primary mitochondrial dysfunction. Clinicians should be aware that serum lactate measurements are susceptible to pre-analytical errors, including prolonged tourniquet time, fist clenching, and delayed processing, which can cause falsely elevated levels. Point-of-care testing minimizes these artifacts and is preferred when available [27, 28]. Bedside lactate testing aids rapid differentiation from other causes of metabolic acidosis. Management includes treating the underlying cause, optimizing perfusion, avoiding mitochondrial toxins (e.g., valproate), and providing metabolic support with dextrose-containing fluids [27, 28].

### Inborn errors of metabolism (IEMs)

IEMs such as organic acidemias (propionic, methylmalonic, isovaleric), maple syrup urine disease (MSUD), and fatty acid oxidation disorders (e.g., MCADD) can present acutely with encephalopathy, ketosis, hypoglycemia, or hyperammonemia. Key biochemical patterns include high anion gap metabolic acidosis, ketonuria, and specific metabolite elevations (e.g., methylmalonic acid, branched-chain amino acids). First-line ED tests should include blood gas, electrolytes, ketones, ammonia, lactate, and urinalysis for reducing substances. Empiric management includes dextrose infusion, carnitine supplementation (100 mg/kg) for suspected organic acidemias, and protein restriction for suspected UCDs [29, 30].

### Electrolyte derangements

Severe hyponatremia (< 125 mEq/L) or hypernatremia (> 155 mEq/L) can cause seizures and cerebral edema. Hyponatremia in the setting of encephalopathy should raise suspicion for syndrome of inappropriate antidiuretic hormone secretion (SIADH), salt-wasting disorders, or adrenal insufficiency. Hypernatremia may indicate diabetes insipidus or dehydration. Rapid but cautious correction is essential to avoid osmotic demyelination. Point-of-care electrolyte testing enables immediate intervention [22, 31]. See Table 2.

**Table 2:** Biochemical patterns and first-line ED tests for metabolic emergencies

Disorder	Key Biochemical Signature	First-Line ED Tests
Hypoglycemia	Blood glucose < 50–60 mg/dL (< 2.8–3.3 mmol/L)	Point-of-care glucose testing, electrolytes, insulin/C-peptide if available
Hyperammonemia	NH₃ >150–200 µg/dL (> 100 µmol/L)	Plasma ammonia (stat), blood gas, liver enzymes, glucose
Lactic Acidosis	Lactate > 2–4 mmol/L, often with high LPR (> 20)	Point-of-care lactate, blood gas, glucose, ketones
Urea Cycle Defects	↑NH₃, respiratory alkalosis, normal anion gap, specific amino acid abnormalities	NH₃, blood gas, plasma amino acids, urine orotic acid
Organic Acidemias	High anion gap metabolic acidosis, ketosis, ↑NH₃, specific organic acids in urine	Blood gas, electrolytes, urine ketones, ammonia, lactate
MSUD	↑↑Leucine, isoleucine, valine; alloisoleucine present; ketonuria	Plasma amino acids, urine ketones, odor detection
Fatty Acid Oxidation Defects	Hypoketotic hypoglycemia, ↑free fatty acids, specific acylcarnitine profile	Glucose, ketones, acylcarnitine profile (if available), liver enzymes
Mitochondrial Disorders	↑Lactate, ↑alanine, elevated LPR, may have normal blood lactate	Lactate, pyruvate, blood gas, plasma amino acids, consider MRS
Electrolyte Imbalances	Na⁺ <125 or > 155 mEq/L, K⁺ abnormalities, acid-base disturbance	Electrolytes, blood gas, glucose, urine electrolytes
DKA	Hyperglycemia, ketonemia, metabolic acidosis, anion gap > 12	Glucose, ketones, blood gas, electrolytes, renal function

**Abbreviations**: LPR = lactate-to-pyruvate ratio; MSUD = maple syrup urine disease; MRS = magnetic resonance spectroscopy; DKA = diabetic ketoacidosis

## Neuro-critical emergencies in the ED

Neuro-critical emergencies in children encompass conditions such as acute encephalopathy, metabolic stroke, status epilepticus from metabolic triggers, and mitochondrial disease crises. These disorders often present without fever or meningism, making them easy to overlook in the ED. Distinguishing them from infectious or structural causes requires vigilance for red flags, bundled metabolic screening, and early EEG when indicated [32, 33].

### Acute encephalopathy

Metabolic encephalopathies often present acutely in previously well children with nonspecific prodromes (vomiting, poor feeding, lethargy) or with fluctuating consciousness lacking focal deficits. Red flags include episodic deterioration after fasting/minor illness, unexplained hyperammonemia, lactic acidosis, hypoglycemia, multisystem signs (hepatopathy, cardiomyopathy), peculiar odors (e.g., maple syrup), and family history of unexplained infant deaths, SIDS, or developmental regression [34, 35]. Key historical red flags include developmental delay or regression (suggesting underlying IEM), family history of unexplained infant deaths or SIDS (raising concern for fatty acid oxidation disorders), consanguinity, and previous pregnancy losses (suggesting possible inherited metabolic conditions) [4, 8]. Rapid ammonia and lactate testing can prevent missed diagnoses [34, 35].

### Metabolic stroke and stroke-like episodes

Mitochondrial disorders such as MELAS (Mitochondrial Encephalopathy, Lactic Acidosis, and Stroke-like episodes) cause stroke-like episodes due to energy failure rather than vascular occlusion. These present with acute focal deficits, seizures, and encephalopathy, often triggered by infection or stress. MRI may show cortical diffusion restriction not conforming to vascular territories. Management includes arginine supplementation, seizure control, and avoidance of mitochondrial toxins [36, 37].

### Status epilepticus from metabolic triggers

Status epilepticus (SE) can be triggered by metabolic derangements such as hypoglycemia, hyponatremia, or IEMs. Nonconvulsive status epilepticus (NCSE) is particularly elusive, presenting as prolonged altered consciousness with minimal motor activity. While NCSE should be considered in any child with unexplained altered consciousness, it is most common in those with known epilepsy or underlying brain injury. In previously healthy children with negative neuroimaging, the yield of EEG for NCSE is lower; in such cases, particularly in resource-limited settings, a diagnostic trial of a benzodiazepine or levetiracetam with careful monitoring of clinical response may be a pragmatic approach before pursuing urgent EEG [38, 39]. Bedside observation alone is unreliable; urgent EEG is diagnostic when available. Continuous EEG monitoring is recommended in comatose children with unexplained encephalopathy, particularly those with refractory seizures or status epilepticus, to detect nonconvulsive seizures [39, 40]. Treatment involves anti-seizure medications alongside correction of underlying metabolic disturbances. Valproate should be avoided in patients with suspected IEMs, particularly mitochondrial disorders or urea cycle defects, as it can precipitate or worsen hyperammonemia and metabolic decompensation [40].

### Mitochondrial crises

**Fig. 2:**
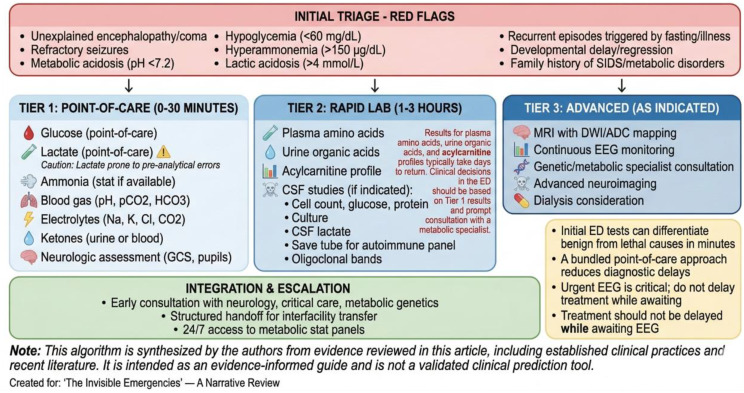
Algorithm for neuro-critical diagnostic flow in the pediatric ED (author-proposed synthesis of published guidance)

Mitochondrial disease crises occur when energy demand outstrips supply during fever, fasting, infection, or anesthesia. Presentations include encephalopathy, refractory seizures, stroke-like episodes, lactic acidosis, and multiorgan dysfunction. Early supportive measures include aggressive temperature control, avoidance of prolonged fasting, cautious medication selection, and early specialist involvement. Point-of-care lactate and glucose testing are essential [41, 42]. See Fig. 2.

## Overlapping syndromes

Children often present to emergency departments with acute neurological symptoms that conceal a complex interaction between neurological and metabolic dysfunction. This overlap, termed **dual pathophysiology**, reflects conditions where metabolic instability amplifies neurological injury, and primary neurological insults trigger secondary metabolic failure. Examples include urea cycle disorders, hypoxic-metabolic encephalopathies, and mitochondrial crises. Early recognition and parallel stabilization of both systems are essential to prevent diagnostic masking and improve outcomes [43, 44].

### Pathophysiological convergence

At the cellular level, both metabolic and neurologic crises converge on disruption of ATP synthesis. Energy failure impairs ionic gradients, triggering neuronal depolarization, cytotoxic edema, and excitotoxicity. Mitochondrial dysfunction serves as a central link—either as a primary defect in metabolic encephalopathies or as a secondary consequence of hypoxia or inflammation. Hypoxia drives anaerobic glycolysis, leading to lactate accumulation and metabolic acidosis, which further inhibits oxidative phosphorylation. Conversely, metabolic crises such as urea cycle defects can induce cerebral edema and secondary hypoxia by disrupting ammonia clearance and vascular autoregulation [45, 46].

### Clinical presentation and diagnostic challenges

Overlap syndromes often present with seizures, altered consciousness, hypotonia, and abnormal movements. Early laboratory or imaging results may appear nonspecific, fostering false reassurance. Metabolic disorders can resemble hypoxic, ischemic, or infectious encephalopathies, contributing to diagnostic uncertainty. Subtle metabolic clues such as respiratory alkalosis in urea cycle defects are easily missed when neurological symptoms dominate. Recurrent encephalopathy following fasting or minor infections, marked elevations in ammonia or lactate disproportionate to the clinical picture, and characteristic MRI findings (e.g., basal ganglia or subcortical signal abnormalities) should prompt consideration of an underlying metabolic disorder [47, 48].

### Management principles

Management requires **parallel stabilization** addressing metabolic and neurologic disturbances simultaneously rather than sequentially. Key interventions include:

- Immediate measurement of glucose, ammonia, lactate, blood gases, electrolytes, and liver enzymes.
- Early consultation with neurology, critical care, and metabolic specialists.
- Prompt reduction of ammonia levels (protein restriction, nitrogen scavengers, dialysis if severe).
- Fluid therapy that supports cerebral perfusion while avoiding exacerbation of cerebral edema.
- Avoidance of medications that may aggravate metabolic dysfunction (e.g., valproate in suspected mitochondrial disorders or urea cycle defects).
- Nutritional management to prevent catabolism and support metabolic stability [49, 50].

See Table 3.

**Table 3:** Mixed Presentations and differential cues in overlap syndromes [45–50]

Clinical Scenario	Metabolic Features	Neuro-Critical Features	Key Differentiating Clues
Acute Encephalopathy + ↑NH₃	Hyperammonemia, respiratory alkalosis	Cerebral edema, seizures, ↑ICP	Central edema on MRI sparing thalami (UCD) vs. diffuse edema (HIE)
Seizures + Lactic Acidosis	↑Lactate, metabolic acidosis	EEG seizures, altered consciousness	Elevated LPR > 25 suggests mitochondrial dysfunction; check for stroke-like episodes on MRI
Coma + Hypoglycemia	Low glucose, ketotic/hypoketotic	Nonconvulsive seizures on EEG	Hypoketotic hypoglycemia suggests FAOD; check acylcarnitine profile
Stroke-like Episode + No Vascular Occlusion	Normal angiography, ↑lactate	Focal deficits, cortical DWI changes	DWI changes not conforming to vascular territory (MELAS)
Encephalopathy + Liver Dysfunction	Elevated transaminases, coagulopathy	Cerebral edema, asterixis	Check ammonia early; consider Reye’s, mitochondrial hepatopathy, or Wilson disease
Recurrent Episodes + Trigger by Illness	Metabolic decompensation with fasting/illness	Encephalopathy, ataxia, vomiting	Family history, previous similar episodes, specific biochemical patterns during crisis

Abbreviations: UCD = urea cycle disorder; HIE = hypoxic-ischemic encephalopathy; LPR = lactate-to-pyruvate ratio; FAOD = fatty acid oxidation disorder; DWI = diffusion-weighted imaging; MELAS = mitochondrial encephalopathy, lactic acidosis, and stroke-like episodes; EEG = electroencephalogram; ↑ICP = increased intracranial pressure

## Diagnostic algorithms

**Fig. 3:**
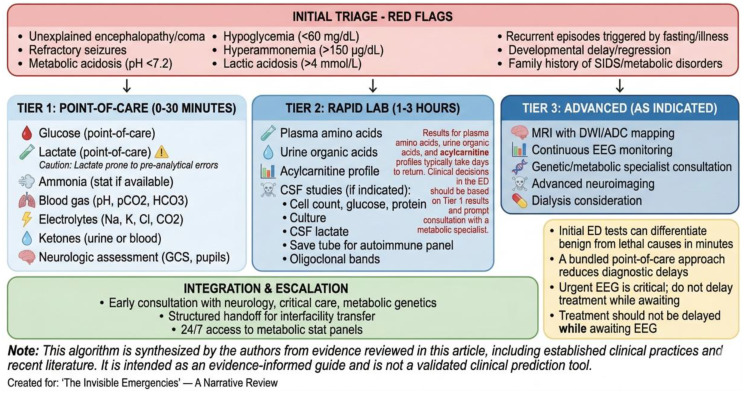
ED diagnostic algorithm for pediatric metabolic and neuro-critical emergencies (author-proposed framework)

**Fig. 4:**
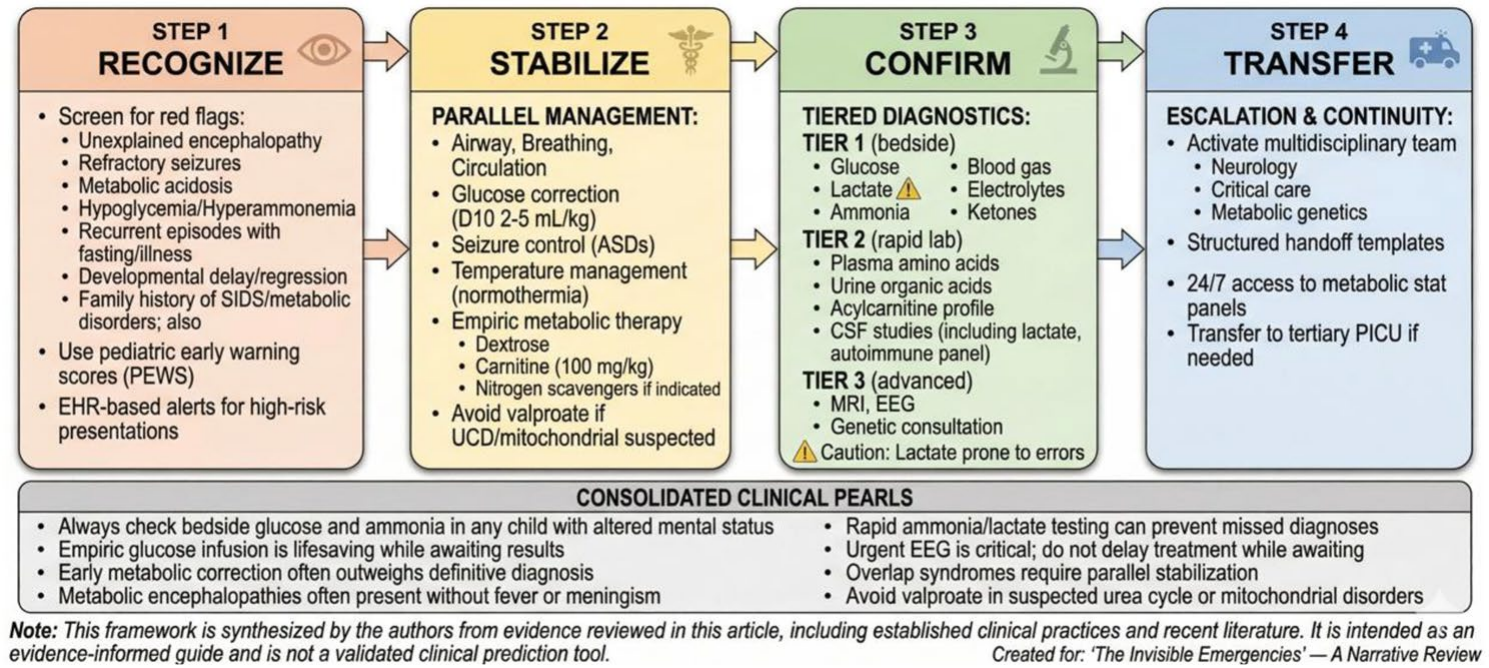
“Early Recognition and Escalation” clinical framework (author-proposed model)

A structured, stepwise diagnostic approach is essential in the pediatric ED to rapidly differentiate benign from life-threatening causes of altered mental status or seizures. The algorithms presented in this section and in Figs. 2, 3 and 4 represent an author-proposed synthesis of published guidelines, consensus recommendations, and clinical experience, rather than formally validated instruments. They are intended as educational tools to guide clinical reasoning and should be adapted to local resources and protocols. Combining symptom triage with biochemical screening and point-of-care tools can significantly reduce time to diagnosis and treatment initiation [51, 52].

### Initial triage and red-flag identification

Upon presentation, clinicians should assess for red flags that warrant immediate metabolic and neuro-critical evaluation:

- Unexplained encephalopathy or coma.
- Refractory seizures.
- Metabolic acidosis (pH < 7.2, elevated anion gap).
- Hypoglycemia (< 60 mg/dL).
- Hyperammonemia (> 150 µg/dL in older children, > 200 µg/dL in neonates).
- Lactic acidosis (lactate > 4 mmol/L).
- Recurrent episodes triggered by fasting/illness.
- Family history of metabolic disorders, unexplained infant deaths, SIDS, or consanguinity.
- Developmental delay or regression [53, 54].

### Point-of-care testing bundle

Within the first 15–30 min, the following tests should be performed at the bedside:

- Glucose (point-of-care glucose testing).
- Lactate (point-of-care lactate meter).
- Ammonia (if available via point-of-care or stat lab).
- Blood gas (pH, pCO2, HCO3).
- Electrolytes (Na, K, Cl, CO2).
- Ketones (urine or blood).
- Basic neurological assessment (Glasgow Coma Scale, pupil reactivity) [55, 56].

### Imaging and neurophysiology

- Non-contrast head CT: if trauma, hemorrhage, or acute hydrocephalus is suspected.
- Bedside ocular ultrasound to measure optic nerve sheath diameter (ONSD) is an emerging point-of-care tool for detecting increased intracranial pressure in children, though its use requires training and validation in pediatric populations [57].
- Urgent EEG should be considered in children with unexplained depressed consciousness, particularly those with known epilepsy, risk factors for seizures, or subtle motor phenomena. In previously healthy children with normal neuroimaging, EEG may be deferred as a second-tier study unless clinical suspicion remains high. If EEG is obtained, simultaneous consultation with pediatric neurology is recommended for interpretation and guidance on further management [39, 58].
- Given the resource burden of obtaining urgent EEG, its use should be targeted to patients with the highest pretest probability of NCSE.
- MRI with diffusion-weighted imaging (DWI) and ADC mapping: when feasible, to differentiate cytotoxic from vasogenic edema and identify metabolic stroke patterns [14, 58].

### Tiered laboratory approach

- **Tier 1 (within 30 min)**: Glucose, electrolytes, renal/liver panels, lactate, ammonia, blood gas, urine ketones.
- **Tier 2 (within 1–3 h)**: Plasma amino acids, urine organic acids, acylcarnitine profile, CSF studies (if infection/autoimmune encephalitis suspected), specific metabolic markers (e.g., homocysteine, pyruvate) [3, 59].

Clinicians should note that Tier 2 tests such as plasma amino acids and urine organic acids typically require several days for results and do not guide immediate ED management. If hyperammonemia or ketosis is identified, early consultation with a metabolic specialist (if available) is recommended to guide empiric therapy and confirmatory testing while awaiting results [3, 59].

When lumbar puncture is performed, CSF studies should include cell count, protein, glucose, Gram stain and culture, and, where available, CSF lactate, paired serum and CSF glucose, and oligoclonal bands. In suspected autoimmune encephalitis (subacute onset of psychiatric symptoms, movement disorders, seizures), send CSF for autoimmune encephalitis panel (NMDA-R, AMPA-R, GABA-B-R, LGI1, CASPR2 antibodies) and paired serum autoantibodies. MRI brain with contrast may show T2/FLAIR hyperintensities in mesial temporal lobes or multifocal areas (ADEM) [14, 33].

### Integration with clinical decision support

Electronic health record (EHR)-embedded clinical pathways can prompt clinicians to order bundled tests when red flags are identified. Simulation training and e-learning modules improve ED team performance in recognizing and managing metabolic and neuro-critical emergencies [24, 60]. See Fig. 3.

## Management and stabilization

Early recognition and stabilization of metabolic and neurocritical disorders in the pediatric ED are essential because these conditions can rapidly progress to life-threatening decompensation. Fundamental priorities include airway protection, glucose homeostasis, fluid resuscitation, seizure control, and temperature regulation. A bundled approach implementing evidence-based interventions simultaneously rather than sequentially is key to optimizing outcomes [35, 61].

### Airway management

The pediatric airway presents unique anatomical challenges. Positioning with a shoulder roll helps maintain a neutral airway. Endotracheal intubation is indicated for Glasgow Coma Scale (GCS) ≤ 8, loss of airway reflexes, or signs of cerebral herniation. Sedation and neuromuscular blockade should be used carefully to prevent intracranial pressure (ICP) spikes. Hyperventilation should be avoided unless there are active signs of herniation, with a goal of maintaining normocapnia (PaCO2 35–45 mmHg) [62, 63].

### Breathing and ventilation

Goal: SpO2 92–94%, normocapnia. Avoid prophylactic hyperventilation as it reduces cerebral blood flow. Brief hyperventilation is reserved only for acute signs of cerebral herniation [63].

### Circulation and fluid management

Maintain euvolemia and avoid hypotension. Isotonic crystalloids (e.g., 0.9% normal saline) are recommended for initial resuscitation, especially in traumatic brain injury (TBI) or cerebral edema risk. Balanced solutions (e.g., Lactated Ringer’s) may be preferred in septic shock or diabetic ketoacidosis (DKA) with cerebral edema to avoid hyperchloremic acidosis. For refractory intracranial hypertension, osmotherapy with hypertonic saline (3%) or mannitol is indicated [64, 65].

### Glucose control

Hypoglycemia (< 50–60 mg/dL) must be treated immediately with 2–5 mL/kg of 10% dextrose. Stress hyperglycemia is common in TBI and sepsis; tight glycemic control is not recommended due to hypoglycemia risk. Treat persistent hyperglycemia (> 180–200 mg/dL) cautiously with insulin infusions [66, 67].

### Seizure control

Status epilepticus requires rapid termination. First-line: benzodiazepines (IV lorazepam 0.1 mg/kg, IM/IN midazolam 0.2–0.5 mg/kg). Second-line: fosphenytoin (20 mg/kg PE), levetiracetam (40–60 mg/kg IV, max 3000 mg), or phenobarbital (15–20 mg/kg IV, may repeat 5–10 mg/kg). Valproate should be avoided in patients with suspected IEMs, particularly mitochondrial disorders or urea cycle defects, as it can precipitate or worsen hyperammonemia and metabolic decompensation. Refractory SE may require continuous infusions (midazolam, pentobarbital) with intubation and EEG monitoring [20, 68].

### Temperature control

Fever increases cerebral metabolic demand; maintain normothermia (36–37.5 °C) with antipyretics and surface cooling. Therapeutic hypothermia (32–34 °C) is reserved for specific contexts (e.g., post-cardiac arrest). Manage shivering with stepwise pharmacological intervention [69, 70].

### Early correction over delayed diagnosis

In suspected metabolic crisis, immediate intervention outweighs definitive diagnosis. Administer glucose, anti-seizure medications, and correct acidosis early to prevent irreversible neuronal injury. Empiric metabolic protocols (dextrose, carnitine, nitrogen scavengers) should be initiated when IEM is suspected [44, 45].

### Escalation to specialists

Prompt referral to pediatric intensive care, neurology, and metabolic genetics is critical when standard interventions fail or when advanced monitoring/therapies are needed. Continuous EEG, dialysis, and specialized pharmacotherapy require tertiary care support [46, 71]. See Table 4.

**Table 4:** Emergency stabilization summary [20–71]

Parameter	Goal / Target	Intervention / Management
Airway	Maintain patency and neutral position	Positioning: shoulder roll for pediatric anatomy. Intubation if GCS ≤ 8, loss of reflexes, or herniation signs
Breathing	SpO₂ 92–94%, normocapnia (PaCO₂ 35–45 mmHg)	Avoid prophylactic hyperventilation. Brief hyperventilation only for acute herniation
Circulation	Euvolemia, avoid hypotension (SBP >5th percentile for age)	Isotonic crystalloids (NS or balanced). 10–20 mL/kg boluses for shock. Avoid hypotonic fluids in cerebral edema risk
Glucose Control	Avoid hypoglycemia (< 60 mg/dL); treat hyperglycemia if persistent > 180–200 mg/dL	Hypoglycemia: 2–5 mL/kg 10% dextrose. Hyperglycemia: cautious insulin infusion, avoid tight control
ICP Management	Maintain ICP < 20 mmHg, CPP > 40–50 mmHg	Head elevation 30°, midline. Osmotherapy: 3% hypertonic saline 2–5 mL/kg or mannitol 0.5–1 g/kg for acute spikes
Seizure Control	Rapid termination of clinical/electrographic seizures	1st line: Lorazepam 0.1 mg/kg IV or midazolam IM/IN. 2nd line: Fosphenytoin 20 mg/kg PE, levetiracetam 40–60 mg/kg, phenobarbital 15–20 mg/kg. Avoid valproate if IEM suspected
Temperature	Normothermia (36–37.5 °C), avoid fever	Aggressive fever control with antipyretics and surface cooling. Manage shivering if therapeutic cooling used

Abbreviations: GCS = Glasgow Coma Scale; SpO₂ = peripheral oxygen saturation; PaCO₂ = partial pressure of carbon dioxide; SBP = systolic blood pressure; NS = normal saline; ICP = intracranial pressure; CPP = cerebral perfusion pressure; IV = intravenous; IM = intramuscular; IN = intranasal; PE = phenytoin equivalents; IEM = inborn error of metabolism

## Challenges in low- and middle-income countries (LMICs)

Pediatric metabolic and neuro-critical emergencies pose particular challenges in LMICs due to limited diagnostic resources, training gaps, and delayed referrals. These barriers contribute to higher morbidity and mortality. However, innovative models such as telemedicine, regional metabolic centers, and basic point-of-care toolkits can help bridge these gaps [72, 73].

### Diagnostic delays and resource limitations

Many LMIC EDs lack access to rapid metabolic testing (ammonia, lactate, amino acids, organic acids) and neurodiagnostic tools (EEG, MRI). Laboratory turn-around times can exceed 24–48 h, delaying life-saving interventions. Additionally, limited availability of specialized pediatric neurologists, intensivists, and metabolic geneticists exacerbates management challenges [74, 75].

### Training and awareness gaps

Frontline healthcare providers in LMICs may have limited exposure to rare metabolic disorders, leading to low clinical suspicion. Educational programs focusing on red-flag recognition, basic metabolic screening, and stabilization protocols are essential. Simulation-based training and telehealth consultations can enhance local capacity [76, 77].

### Innovative solutions

- **Point-of-Care Testing Kits**: Low-cost glucometers, lactate meters, and ammonia strips can enable rapid bedside screening.
- **Telemedicine Networks**: Connecting district hospitals with tertiary centers for real-time consultation and guidance.
- **Regional Metabolic Registries**: To track incidence, outcomes, and resource needs.
- **Simplified Diagnostic Algorithms**: Tailored to available resources, emphasizing clinical cues and basic labs [78, 79].

### Resource-stratified management strategies

- **Basic Setting (minimal labs)**: Focus on glucose, electrolytes, clinical red flags, empiric dextrose/fluids, and early referral.
- **Intermediate Setting (some labs)**: Add lactate, ammonia, ketones, blood gas, and basic anti-seizure medications.
- **Advanced Setting (full labs + neurodiagnostics)**: Comprehensive metabolic panels, EEG, MRI, and specialist management [80, 81].

See Table 5.

**Table 5:** Resource-stratified management strategies for LMICs [76–81]

Setting	Available Diagnostic Resources	Key Actions	Referral Triggers
**Basic (Minimal)**	Bedside glucose, clinical exam only	– Empiric dextrose for altered mental status – IV fluids, seizure control with available ASMs – Early referral if no improvement	– Persistent coma – Refractory seizures – Suspected metabolic disorder
**Intermediate**	Glucose, electrolytes, blood gas, lactate, basic microscopy	– Add lactate/ketone testing – Correct acidosis/electrolytes – Use ammonia strips if available – Start empiric metabolic therapy	– Hyperammonemia – Severe acidosis – Need for advanced imaging/EEG
**Advanced**	Full metabolic panel, ammonia, amino acids, urine organic acids, EEG, MRI	– Comprehensive tiered workup – Specialist consultation (neurology/metabolism) – Continuous EEG monitoring if available – Dialysis if needed	– Transfer to tertiary PICU for ongoing care

Abbreviations: ASMs = anti-seizure medications; IV = intravenous; EEG = electroencephalogram; MRI = magnetic resonance imaging; PICU = pediatric intensive care unit

## Proposed clinical framework

A unified clinical framework integrating early warning, triage, and diagnostic panels is essential for improving outcomes in pediatric metabolic and neuro-critical emergencies. The proposed model follows a **“Recognize–Stabilize–Confirm–Transfer”** sequence, promoting structured decision-making under uncertainty [62, 63]. This framework represents an author-proposed synthesis of clinical experience and published guidance, intended as an educational tool rather than a validated protocol.

### Step 1: Recognize (Early Warning Signs)

- Screen for red flags: unexplained encephalopathy, refractory seizures, metabolic acidosis, hypoglycemia, hyperammonemia, recurrent episodes triggered by fasting/illness.
- Key historical red flags: developmental delay/regression, family history of unexplained infant deaths/SIDS, consanguinity, previous pregnancy losses.
- Use validated pediatric early warning scores (PEWS) integrated with metabolic triggers where available.
- Implement EHR-based alerts for high-risk presentations [82, 83].

### Step 2: Stabilize (Parallel Management)

- Simultaneously address neurologic and metabolic priorities:
  - Airway, breathing, circulation.
  - Glucose correction, seizure control, temperature management.
  - Empiric metabolic therapy (dextrose, carnitine, nitrogen scavengers) when indicated.
- Avoid anchoring on a single diagnosis; maintain dual-pathway thinking [80, 84].

### Step 3: Confirm (Tiered Diagnostics)

- **Tier 1 (bedside)**: Glucose, lactate, ammonia, blood gas, electrolytes, ketones.
- **Tier 2 (rapid lab)**: Plasma amino acids, urine organic acids, acylcarnitine profile, CSF studies (cell count, protein, glucose, culture, lactate, oligoclonal bands, autoimmune panel when indicated).
- **Tier 3 (advanced)**: MRI, EEG (with neurology consultation for interpretation), genetic/metabolic specialist consultation [81, 82].

### Step 4: Transfer (Escalation and Continuity)

- Early activation of multidisciplinary teams (neurology, critical care, metabolic genetics).
- Use structured handoff templates to ensure communication during interfacility transfer.
- Ensure 24/7 access to metabolic stat panels and urgent EEG where resources permit [85, 86].

### Implementation strategies

- Develop and disseminate ED-specific clinical pathways.
- Train ED staff through simulations and e-learning modules.
- Integrate biomarker-informed scoring within AI triage systems for risk stratification (emerging/investigational) [87, 88].

See Fig. 4.

## Future directions and research gaps

The convergence of artificial intelligence (AI), metabolic biomarkers, pediatric-specific scoring systems, and genomic/registry data presents a powerful opportunity to modernize pediatric triage and improve outcomes for metabolic and neuro-critical emergencies. However, challenges related to ethics, data quality, and implementation remain [85, 86].

### AI-supported triage systems

AI and machine learning (ML) tools are emerging as potential adjuncts to enhance pediatric triage by integrating structured vital signs with unstructured data (triage notes, presenting complaints) to identify clinically meaningful patterns. Ensemble models have demonstrated promising accuracy in research settings for classifying urgency, though these approaches remain investigational and require validation in diverse pediatric populations [87, 88]. Emerging research suggests that AI clustering methods may in the future help identify children at risk of rapid deterioration despite normal initial vitals, potentially reducing delayed recognition of sepsis or metabolic instability [87, 88].

### Biochemical biomarkers in triage

Incorporating metabolic biomarkers (lactate, albumin, glucose, LDH) into triage algorithms offers an objective foundation for early risk stratification. Composite ratios such as LDH/albumin have shown independent predictive value for mortality in critically ill children in selected studies. These inexpensive, rapid tests are ideal for real-time integration across high- and low-resource settings, though further validation is needed [89, 90].

### Genetic screening and registry data

Integrating genomic data—through targeted genetic screening or EHR-linked genomic flags—can identify at-risk subgroups (e.g., hyper-inflammatory sepsis phenotypes, adrenal insufficiency predisposition). Large pediatric registries facilitate ML-derived subphenotypes, enabling next-generation triage systems that assign acuity based on underlying biological signature rather than presentation alone. These approaches are currently in early stages of development [84, 91].

### Implementation challenges

Practical implementation requires rapid-turnaround biochemical assays, automated data ingestion into EHRs, and real-time AI-driven decision engines. Ethical considerations include data privacy, algorithmic bias, and equity in access. Future research should focus on validating hybrid biomarker-AI tools in diverse pediatric populations and settings [84, 91].

## Conclusion

Metabolic and neuro-critical disorders in children represent “invisible emergencies” that are frequently missed or delayed in ED recognition due to nonspecific presentations, low clinical suspicion, and limited diagnostic resources. This review has synthesized current evidence to address the research question: *Which metabolic and neuro-critical disorders in pediatric patients are under-recognized in emergency departments*,* and what evidence-based strategies can enhance early diagnosis*,* stabilization*,* and outcomes?*

Key takeaways include:

- **High-risk conditions**: IEMs (urea cycle defects, mitochondrial disorders, organic acidemias), endocrine crises, NCSE, autoimmune encephalitis, ADEM, and metabolic strokes.
- **Red flags**: Unexplained encephalopathy, refractory seizures, metabolic acidosis, hypoglycemia, hyperammonemia, recurrent episodes triggered by fasting/illness, developmental regression, family history of unexplained deaths or SIDS.
- **Diagnostic strategies**: Bundled point-of-care testing (glucose, lactate, ammonia, blood gas), tiered laboratory panels, urgent EEG in selected patients, early imaging, and CSF studies when indicated.
- **Management principles**: Parallel stabilization of metabolic and neurologic systems, empiric therapy (including glucose, carnitine, nitrogen scavengers when indicated), avoidance of valproate in suspected IEMs, and prompt escalation to specialists.
- **Framework implementation**: The proposed “Recognize–Stabilize–Confirm–Transfer” model provides a structured approach for ED clinicians, though it requires adaptation to local resources and validation in diverse settings.

Enhanced awareness, standardized pathways, interdisciplinary collaboration, and technological innovations (AI, biomarkers, telemedicine) are critical to reducing diagnostic delays and improving outcomes. Continued refinement of integrated ED workflows, coupled with training and resource allocation—especially in LMICs—will strengthen our ability to deliver timely, effective care for these complex, life-threatening presentations.

## Data Availability

Data sharing is not applicable to this article as no new data were created or analyzed.
